# Atypical Findings of Focal Nodular Hyperplasia with Gadoxetic Acid (Gd-EOB-DTPA)-Enhanced Magnetic Resonance Imaging

**DOI:** 10.5812/iranjradiol.9269

**Published:** 2014-01-30

**Authors:** Jung-Hee Yoon, Ji-Yeon Kim

**Affiliations:** 1 Department of Radiology, Haeundae Paik Hospital, Inje University College of Medicine, Jwa-dong, Haeundae-gu, Busan, Korea; 2 Department of Pathology, Haeundae Paik Hospital, Inje University College of Medicine, Jwa-dong, Haeundae-gu, Busan, Korea

**Keywords:** Liver Neoplasms, Focal Nodular Hyperplasia, DTPA

## Abstract

We report two cases of focal nodular hyperplasia in patients following gadoxetic acid (Gd-EOB-DTPA)-enhanced magnetic resonance imaging confirmed with histopathology. These cases showed an atypical pattern during the delayed-hepatobiliary phase after the injection of gadoxetic acid. One case showed a total defect, and the other showed a peripheral ring-like enhancement without a visible central scar, mimicking hepatocellular carcinoma. The pathologic examination demonstrated that the two lesions were focal nodular hyperplasia.

## 1. Introduction

Focal nodular hyperplasia (FNH) is the second most common benign lesion of the liver after hemangioma (1). Because FNH has no malignant potential or life-threatening complications such as hepatocellular adenoma, further intervention or surgical resection is not needed when the diagnosis is confirmed (2). The characteristic radiologic findings of FNH have been well documented, but the exact distinction of FNH from other hypervascular hepatic tumors is not easy, especially in cases of small lesions. Gadoxetic acid (Gd-EOB-DTPA, Primovist®, Bayer Schering Pharma AG, Berlin, Germany) is a hepatocyte-specific magnetic resonance (MR) contrast agent that is increasingly used for liver MR imaging. Gadoxetic acid is actively taken up by hepatocytes and excreted along the bile duct and kidney. It is known to be specific for the diagnosis of FNH, showing hyperintense or isointense regions compared to the liver during the delayed hepatobiliary phase (3, 4). Several atypical imaging findings have been reported for FNH (5), but most are CT-based imaging findings, and there are only a few reports concerning atypical findings in hepatocyte-specific MR contrast-enhanced imaging. Here, we describe two cases of FNH presenting with atypical hepatobiliary phase findings on gadoxetic acid-enhanced MR imaging.

## 2. Case presentation

### 2.1. Case 1

**Figure 1.**
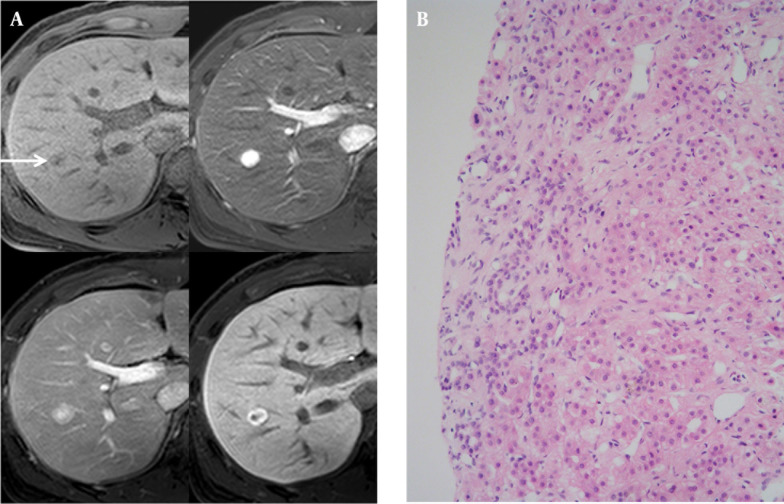
A 29-year-old man with underlying chronic hepatitis B and an incidentally detected hepatic nodule. A, On T1-weighted gradient-echo imaging (TR/TE: 3.6/1.4), there was a subtle low signal intensity nodule (upper left, arrow). On gadoxetic acid-enhanced MR imaging, the nodule demonstrated early homogeneous intense enhancement (upper right), subtle peripheral washout during the portal venous phase (lower left), and ring-like peripheral enhancement with a central washout pattern on 20 minutes delayed hepatobiliary phase (lower right). B, Histopathology reported proliferating hepatocytes and bile ductules with fibrous septae on the border compatible with focal nodular hyperplasia.

A 29-year-old man with underlying chronic hepatitis B visited our out-patient hospital clinic. His serum alpha-fetoprotein level was within normal limits (3.05 ng/mL, normal limit: ~7.0 ng/mL). An abnormal 1.3 cm mixed echoic hepatic nodule was found in the right lobe incidentally on routine screening ultrasonography. The hepatic nodule had a peripheral hypoechoic halo, mimicking a malignant nodule. Multi-detector computed tomography (MDCT) was performed to characterize the focal liver lesion. The hepatic nodule showed complete enhancement during the early arterial phase of MDCT that then faded to iso-attenuation during the portal venous phase and the delayed phase without definite washout of the contrast enhancement. We proposed several possible diagnoses for this incidental nodule, including a well-differentiated hepatocellular carcinoma (HCC) and a high-grade dysplastic nodule due to the background of chronic hepatitis. FNH and hepatic adenoma were also included in the differential diagnosis. On T2-weighted imaging (T2WI) of MR, the nodule showed a high signal intensity (SI) and mild diffusion restriction on diffusion- weighted imaging (DWI, diffusion b-factor, 800) and apparent diffusion coefficient imaging. On gadoxetic acid-enhanced MR imaging, the mass demonstrated early intense homogeneous enhancement and subtle peripheral washout during the portal venous phase and ring-like peripheral enhancement with a central washout during the equilibrium phase and hepatobiliary phase (20 minutes, Figure 1 A). We hypothesized that the ring-like peripheral enhancement was suggestive of the capsule of a HCC. Ultrasound-guided biopsy was performed, and the biopsy specimens were grossly visualized as yellowish gray soft tissue and proliferating bile ductules with fibrous stroma and normally proliferating hepatocytes (Figure 1 B), which was histopathologically indicative of FNH. The nodule was stable over the 18 months of follow-up. 

### 2.2. Case 2

**Figure 2.**
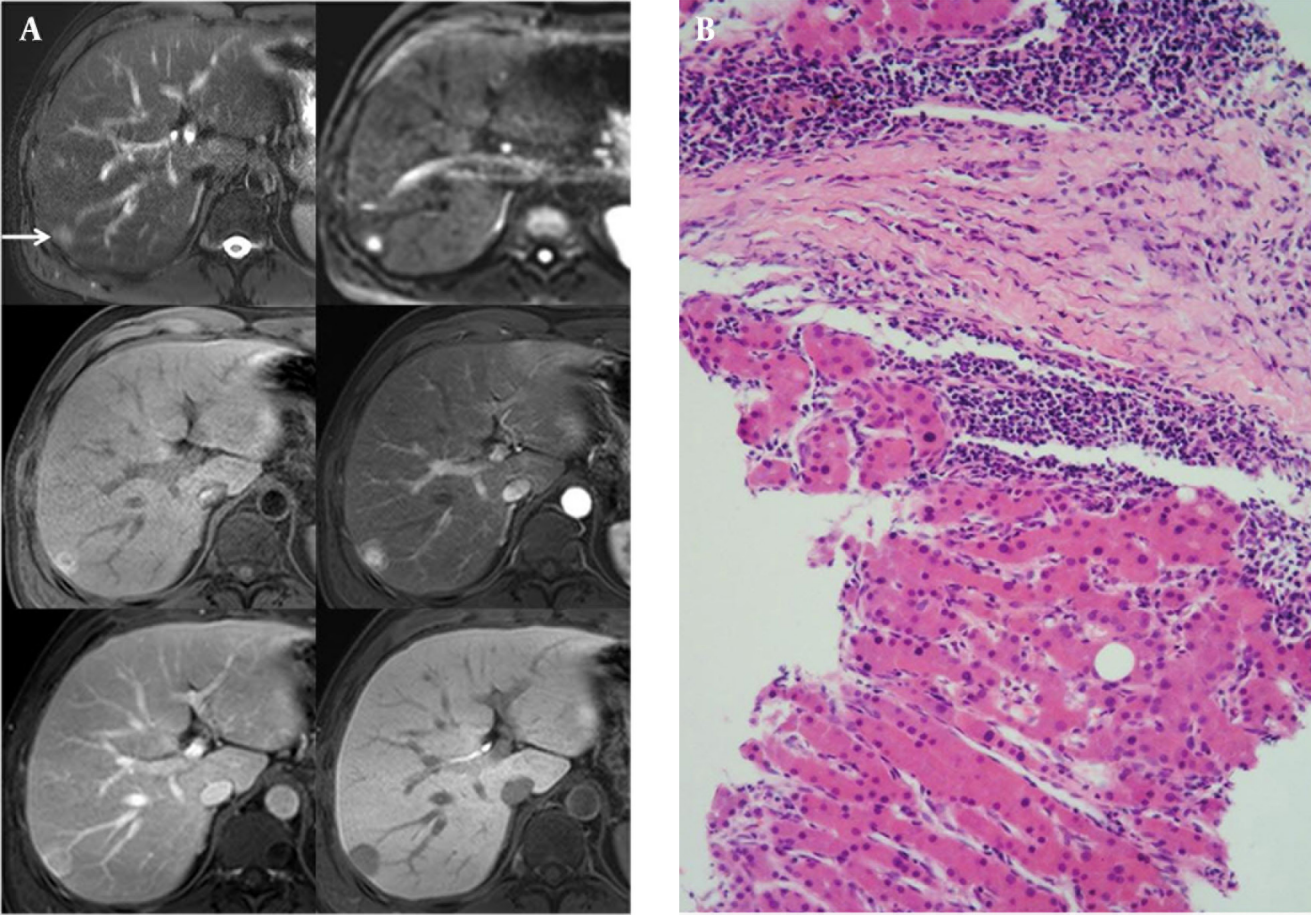
A 39-year-old man with a history of heavy alcoholism. A, T2-weighted fast-spin echo imaging (TR/TE: 3646.3/107. 0, upper left, arrow), and diffusion weighted imaging (b-factor, 800, upper right) showed a high signal intensity nodule in the subcapsular portion of the right hepatic lobe. On T1-weighted gradient-echo imaging (TR/TE: 3.6/1.4), there was a high signal-intensity nodule with a peripheral halo (middle left). On gadoxetic acid-enhanced MR imaging, the nodule was visualized with early homogeneous enhancement (middle right), washout during the equilibrium phase (lower left), and complete perfusion defect on the 20 minutes delayed hepatobiliary phase (lower right). B, On histopathology (HE stain, high-power field [×200], a fibrous septum was observed in the tumor nodule exhibiting signs of lymphocytic infiltration. The hepatocytes formed one or two cell thick trabecular cords and were cytologically benign proliferating hepatocytes compatible with a diagnosis of focal nodular hyperplasia.

A 39-year-old man with a 5-year-history of alcoholism visited our hospital presenting with fatigue and dizziness. A well-defined, hypoechoic, well-encapsulated mass in the subcapsular portion of the right hepatic lobe approximately 2.4 cm × 1.5 cm in size was found during the ultrasound examination. Spotty intratumoral vessels were noted on color Doppler imaging. On MDCT, we observed homogeneous mild enhancement during the arterial phase and central washout with ring-like enhancement during the portal venous phase that faded during the delayed phase. On T2WI MR imaging, the nodule showed a high SI and a mild diffusion restriction on DWI. On gadoxetic acid-enhanced MR imaging, a well-defined, well-encapsulated, subtle high SI hepatic nodule was observed on T1-weighted imaging (T1WI) showing homogeneously intense enhancement during the early phase, subtle washout during the delayed phase and a low signal perfusion defect during the hepatobiliary phase (Figure 2 A). We considered the possibility of an expanding nodular HCC, most probably because this mass showed a complete defect during the hepatobiliary phase. Ultrasound-guided biopsy was performed for confirmation, and the histopathologic reports showed hepatocellular nodule with fibrovascular septae favoring FNH (Figure 2 B). This nodule showed no change over the 25 months of follow-up CT. 

## 3. Discussion

FNH is found in approximately 8% of all primary liver tumors, making it the second most common lesion after hemangioma (6). The prevalence of FNH is up to 3% and it affects all ages. FNH is most common between the third and fifth decades of life, and women are predominantly affected with a female-to-male ratio of 6 to 8:1 (7). The precise etiology of FNH is not well understood, but the most accepted theory posits a congenital vascular malformation as the trigger event; however, it is thought to arise as a result of larger-than-expected pre-existing spider-like arterial structures with heterogeneous blood flow in the liver cellular architecture, resulting a hyperplastic hepatocytic response (8). FNH is a benign regenerative nodule composed of disorganized growing normal hepatocytes forming an unencapsulated well-defined mass with abnormally structured vessels and bile ducts. Histopathologic confirmation is based on the demonstration of normal-appearing hepatocytes, Kupffer cells, or a central scar and blood vessels centered in nodules and surrounded by fibrous septa containing primitive bile ductules (9). The MR imaging of classic FNH is an iso- or hypointense lesion on T1WI and an iso-or slightly hyperintense lesion on T2WI. Often, the differential diagnosis between FNH and hepatic adenoma can be supported by the presence of characteristic features, especially central scar of FNH or heterogeneous MR SI of hepatic adenoma due to intralesional hemorrhage or fat component (10). FNH rarely has a high SI on T1WI unlike hepatic adenoma. On dynamic contrast-enhanced MR images, FNH usually shows homogeneous enhancement during the arterial phase, compartmentalized by radiate fibrous septae, arising from the nonenhancing central scar. The central scar of FNH usually shows a high SI on T2WI and delayed enhancement because it is mainly a vascular and inflammatory scar. The point of distinction from the central scar of fibrolamellar HCC is that it is hypointense on T1- and T2WI without definite enhancement, suggesting the true fibrous scar that usually shows large, eccentric broad fibrous bands and calcification (11). Approximately, in only 20-35.7% of FNHs, a central low attenuated scar may be observed on imaging (9, 12). The lack of a central scar on preoperative imaging makes diagnosis difficult in almost all patients with non-classical FNH and in some patients with classical FNH. Even a combination of several imaging techniques, including color Doppler imaging, MDCT and MR imaging can definitively prove the diagnosis in up to 50% of FNH patients, especially when the patients present with small FNHs less than 3cm (12). Currently, various liver-specific MR contrast agents have been developed and often used in clinical studies with problem-solving modality (4). There is a class of superparamagnetic iron oxide (SPIO) targeted to the reticuloendothelial system of the liver, the other class of agents targeted to hepatocytes.

On SPIO-enhanced T2WI MR images, FNH is hypointense compared with the normal liver, due to phagocytosis by the reticuloendothelial systems (Kupffer cells), and the central scar is more prominent. Atypical findings of FNH have been reported to include hemorrhage, necrosis, fat accumulation, surface retraction, rapid contrast washout, and the absence of a central scar (5, 13, 14). Usually, FNH is not commonly encapsulated, but there is rather often pseudocapsule formation due to compression by the surrounding liver parenchyma. Inflammatory changes may also lead to an appearance more similar to a true capsule, which is visualized as a hypoechoic halo on ultrasound studies, similar to case 1 in this report. Gadoxetic acid is a newly available hepatobiliary-specific MR contrast agent that can be used for dynamic imaging by the bolus injection method. Regarding its hepatocyte-selective properties, the contrast is concentrated in the liver parenchyma during delayed imaging. It has been reported that it can improve the detection and characterization of focal liver lesions, and it can particularly be used to determine whether a lesion is of hepatocellular origin (3, 4). In many investigations, FNH demonstrates bright enhancement during the arterial phase on gadoxetic acid-enhanced MR images similar to other extracellular gadolinium-based contrast. Unlike hepatic adenoma, FNH showed a delayed hepatocellular accretion of the contrast because the well-rounded bile canalicular systems is not sufficient in FNH for normal bile excretion. Thus, constant hyper- or isointense signal compared with the surrounding liver parenchyma during the hepatobiliary phase is observed in approximately 88-90% of FNH patients (15). Another evaluation on a large group of hepatic lesions composed of 235 FNH or hepatic adenomas revealed that the overall accuracy for the differentiation of FNH from hepatic adenoma was 98.3% (3). While all hepatic adenomas appeared hypointense, only four of 128 relatively small FNHs (3.1%) demonstrated atypical hypointensity on hepatobiliary phase imaging. They also reported histologic findings of these atypical cases without any information about distinct histologic differences from other histologically confirmed FNH that showed typical homogeneous hyperintensity on hepatobiliary phase imaging (3). Up to now, not much is known about the potential reason why some of FNHs are presented with “washout” on the hepatobiliary phase. The mechanism for washout in HCC is related to the comparative concentration of arterial and portal venous blood flow throughout the lesion and the liver (16). Reportedly, central or hepatic veins were accountable for draining blood and no portal vein branches working as draining veins from the lesions were seen (17). In addition, another report has described the presence of early draining that is known to be most frequently associated with HCC and in some of FNHs (18). However, there is confusion about whether the presence of early draining vein and absence of portal vein branches are responsible for “washout” in FNHs. A FNH case of ring-like enhancement similar to case 2 has been reported (19). The authors reported immunohistochemistry findings for the resected specimen, and the hepatocytes in the peripheral areas of the lesion showed strong OATP8 expression, while the hepatocytes nearby the central areas and adjacent to the thin radiating scars were negative for OATP8 expression. OATP8 is a member of the solute carrier organic anion transporter family and it is particularly demonstrated at the basolateral membrane of normal hepatocytes; OATP8 is expressed in pericentral hepatocytes but it is absent in periportal hepatocytes. As the central scar of FNH contains bile ducts, it is rational that hepatocytes nearby the central scar do not express OATP8 similar to periportal hepatocytes (19). They indicated that the central nonenhancing portion is due not only to the existence of the central scar, but also to the deficiency of OATP8 expression in the hepatocytes nearby the central scar. They suggested that the ring-like enhancement in the hepatobiliary phase may be a significant evidence for the diagnosis of small FNHs. We did not need to perform surgical resection because it was diagnosed with ultrasonound- guided biopsy, and did not stain OATP8 immunohistochemical study. But ring-like enhancement in the hepatobiliary phase is commonly visible in the metastases or cholangiocarcinomas due to their fibrous component or in HCC due to the fibrous capsule. At the end, in this report, two FNH patients with atypical findings of gadoxetic acid- enhanced MR imaging have been reported in addition to a review of the literature. One showed a complete defect, and the other showed a peripheral ring-like enhancement without a visible central scar. For definitive diagnosis of FNH, delayed hepatobiliary phase MR imaging does not provide an absolute answer, especially when the tumor diameter is less than 3 cm. These lesions show arterial enhancement and may show washout during the hepatobiliary phase mimicking HCC. Thus, MR imaging using a hepatobiliary-specific contrast agent may result in better delineation of FNH, but atypical features may cause difficulties in definite diagnoses.
